# Assessment of the immunogenicity of residual host cell protein impurities of OsrHSA

**DOI:** 10.1371/journal.pone.0193339

**Published:** 2018-03-07

**Authors:** Naghmeh Abiri, Jianlei Pang, Jiquan Ou, Bo Shi, Xianghong Wang, Sucai Zhang, Yunxia Sun, Daichang Yang

**Affiliations:** 1 State Key Laboratory of Hybrid Rice, College of Life Sciences, Wuhan University, Wuhan, China; 2 Healthgen Biotechnology Co. Ltd., Wuhan, China; 3 JOINN Laboratories, Inc., Beijing, China; Fujian Agriculture and Forestry University, CHINA

## Abstract

Human serum albumin (HSA) is the most abundant protein in human plasma and is widely used at high doses for treating various diseases. Recombinant HSA is an alternative approach to plasma-derived HSA, providing increased safety and an unlimited supply. However, the safety of the residual host cell proteins (HCPs) co-purified with *Oryza sativa* HSA (OsrHSA) remains to be determined. An animal system was used to assess the immunogenicity of OsrHSA and its residual HCPs. Low immunogenicity and immunotoxicity of the residual HCPs at a dose of 25 μg/kg, equivalent to 25 times the clinical dosage of HSA, were observed. An anti-drug-antibody (ADA) analysis revealed that anti-HSA, anti-OsrHSA or anti-HCP antibodies developed with a low frequency in pHSA and OsrHSA treatments, but the titers were as low as 1.0–2.0. Furthermore, the titer and the incidence of the specific antibodies were not significantly different between the pHSA and OsrHSA groups, indicating that OsrHSA presents similar immunogenicity to that of pHSA. More importantly, no cytokines were stimulated after the administration of OsrHSA and the residual HCPs, suggesting that there was no risk of a cytokine storm. These results demonstrated that the residual HCPs from OsrHSA have low immunogenicity, indicating that the rice endosperm is one of the best hosts for plant molecular pharming.

## Introduction

Molecular pharming refers to the use of a host, either plant or animal, for the large-scale production of commercially valuable recombinant proteins. Numerous biologics, vaccines, antibodies and other pharmaceuticals, totaling more than 108 pharmaceutical proteins, have been produced via plant molecular pharming [1–15]. There are at least 16 pharmaceutical proteins in phase I, five in phase II and three in phase III clinical trials [16]. Only two plant-made pharmaceuticals (PMPs), SIgA, also called CaroRx, and the glucocerebrosidase Elelyso, have been approved by regulatory authorities [17,18]. One reason for the lack of regulatory approval is concern regarding the level and population of residual host cell proteins (HCPs) in PMPs, which could produce potential immunogenicity [19].

In general, completely removing residual HCPs from the final pharmaceutical product in downstream processing is a huge challenge. Studies have reported a subset of HCPs, referred to as “difficult to remove,” that strongly interact and co-purify with therapeutic proteins during downstream purification [20]. Indeed, the level and populations of HCPs not only alter the safety but also influence the efficacy of biopharmaceuticals. Therefore, residual HCPs are defined as critical quality attributes (CQAs) because these residues cannot be completely removed, although approaches to minimize the levels of the HCP are employed. One of the major risks of the HCPs in PMPs is their potential immunogenicity. Thus, the characterization of residual HCPs in PMP products as CQAs and the assessment of the immunogenicity through various approaches are of paramount importance. Such assessments include proteomic and biochemical methods [21–23], bioinformatics to directly examine and characterize data sets, literature reviews of published scientific knowledge and peripheral blood mononuclear cells (PBMCs) [24,25]. However, reliable data on the immunogenicity of HCPs are directly obtained through animal/human preclinical and clinical studies [19,26]. Studies on the immunogenicity of residual HCPs through preclinical or clinical trials are sparse, and information on the immunogenicity of the HCPs from PMPs is completely absent.

Human serum albumin (HSA) is the most abundant protein in human plasma [27]. HSA is a soluble, globular, unglycosylated, monomeric multi-domain protein widely used in the clinic to treat numerous conditions, including hypovolemia, hemorrhagic shock, serious burns, surgical blood loss, cardiopulmonary bypass, acute respiratory distress syndrome, hemodialysis, acute liver failure, chronic liver disease, nutrition support, resuscitation, and hypoalbuminemia, and as an adjunctive therapy for cancer chemotherapy and radiotherapy [28–30]. Although recombinant HSA (rHSA) derived from *Oryza sativa* (OsrHSA) has been produced in transgenic rice seed on a large scale [5] and was recently approved for clinical trials in China, the safety of the HCPs from OsrHSA is a critical concern. Thus, it is necessary to assess the safety risk and set a CQA as the risk-based HCP limit of OsrHSA prior to clinical trial.

In the present study, to establish the risk-based HCP limits of OsrHSA, we assessed the immunogenicity and immunotoxicity of OsrHSA and its residual HCPs. The results indicated the low immunotoxicity of the HCPs in the animal system. We did not observe a significant difference in the adverse immune response between plasma-derived HSA (pHSA) and OsrHSA. The immunological analysis of CD4+, CD8+, complement 3 (C3), complement 4 (C4), and the titers and incidences of specific antibodies revealed no significant difference between OsrHSA and pHSA. No cytokines were stimulated after the administration of the HCPs and OsrHSA, suggesting that the risk of a cytokine storm is non-existent should OsrHSA be applied in the clinic. Furthermore, we did not detect IgM or IgE in the HCP and OsrHSA groups. These results supported the hypotheses that human beings have developed tolerance to rice proteins during evolution and that the residual HCPs derived from OsrHSA are safe. These results provide evidence showing that rice seed is an excellent host for plant molecular pharming.

## Materials and methods

### Preparation of HCPs from rice seeds

Approximately 1,500 kg of rice powder derived from the host variety TP309 was used to prepare the HCPs following a standard operation protocol of manufacturing OsrHSA (DMF No. 029648, https://www.fda.gov/drugs/developmentapprovalprocess). Briefly, rice seeds were ground into powder and homogenized in a phosphate buffer (PB) [25 mM PB with 50 mM NaCl (pH 7.5)] at a ratio of 1:5 (wt/vol) at room temperature for 1 h, and the mixture was precipitated for 2 h at pH 5.0. The crude extract was clarified by a filter press skid. The clarified extracts were carried out by three steps of chromatography, i.e. Capto-MMC, Q Sepharose Fast Flow and Phenyl HP (GE Healthcare, www.gelifesciences.com). The final residual HCPs from the final step of the chromatography was collected (Lot No. 201512001) and were concentrated to the appropriate concentration for future use. OsrHSA (Lot No. C201504001) was provided by Healthgen Biotechnology Co. Ltd, Wuhan, China (http://www.oryzogen.net), and its residual HCP content was 1.5 μg/g, as determined by Bradford assay. pHSA (Lot No. 201206A049) was purchased from the Institute of Chengdu Biological Products.

### Animal maintenance conditions

Sprague-Dawley rats (6–7 weeks of age and body weight 176–194 g) were purchased from Beijing Weitong Lihua Experimental Animal Technology Co., Ltd. Beijing, China (Production Certificate No. SCXK 2012 0001). The animals were housed in 485 mm x 350 mm x 200 mm cages (polycarbonate boxes), and the maximum number of animals in each cage was five animals of the same sex. The room temperature and humidity of the cages were 21–27°C and 42% to 70%, respectively. All animal conditions followed the good laboratory practice (GLP) guidance (GB1424.2–2001 and GB14924.3–2010). The animals were used in the experiments to ensure the highest possible standards of animal welfare. The animals in JOINN Laboratories, Inc. were handled as in an ethical way. The best husbandry available to experience a stress-free life and good health was provided, which have been accredited for Assessment and Accreditation of Laboratory Animal Care (AAALAC) by International association. This study was approved by the Institutional Animal Care and Use Committee (IACUC) at JOINN Laboratories, Inc. (Approval No. ACU16-174).

### Dose design and animal grouping

Four groups—OsrHSA, residual HCPs, pHSA (positive control) and PBS (negative control)—were designated. The dose administered to each group was calculated based on the clinical dose of pHSA (10 g/dose/60 kg/person, i.e., a dosage of 0.2 g/kg/day). The doses were designated as 5 g/kg for OsrHSA and pHSA and 25 μg/kg for HCPs, which is equivalent to 25-fold the clinical dose of pHSA. All treatments were intravenously infused via the tail veins at a rate of approximately 4.5 mL/min/kg (Day 1-Day 8) and 4.0 mL/min/kg (Day 9-Day 14). The administration was continual for 14 days at 24-h intervals. After the last administration (Day 14), a 4-week recovery period was set for clinical observation and various analyses.

### T-lymphocyte subset typing

A BD FACSCalibur flow cytometer and Cytometric Bead Array kits were used according to the manufacturer’s instructions (BD Bioscience, San Jose, CA, USA). The data were collected and calculated using the FACP Array software (BD Bioscience, San Jose, CA, USA).

### Cytokine assays

The Meso Scale Discovery kit (Meso Scale Diagnostics, Rockville, MD, USA) was used for cytokine separation following the manufacturer’s instructions. Cytokine levels were determined using a MESOTM QuickPlex SQ 120 (Meso Scale Diagnostics, Rockville, MD, USA).

### C-reactive protein (CRP) assay

A CRP assay kit (Abcam, Lot No. GR268663-1 and GR229360-7) was used following the manufacturer’s instructions. CRP was measured at 450 nM with the SpectraMax® M5/M5e Multimode Plate Reader (Molecular Devices, CA, USA).

### Plasma circulating immune complex assay

A circulating immune complex (CIC) assay kit (LYD, lot No. 201605 and 201606) was used following the manufacturer’s instructions.

### Complement measurement

The complement ELISA kits for C3 (Abcam, Shanghai, China, Cat No. GR241315-6 and GR241315-9) and C4 (Abbexa, Cambridge, UK, Cat No. L701605F112) were used. The measurements were obtained according to the manufacturer’s instructions.

### ELISA for residual HCP-specific antibody

A validated ELISA protocol for the detection of HCP-specific, pHSA-specific and OsrHSA-specific antibodies was developed. In detail, the HCPs (5 mg/mL) were diluted to 20 μg/mL with coating buffer (bicarbonate/carbonate 100 mM), and 100 μL/well was added to the microtiter plate. The microtiter plate was incubated at 4°C overnight and subsequently washed 3 times with 300 μL of PBST (PBS with 0.1% Tween 80) for 5 min each. Then, 300 μL of blocking solution (PBS, 5% defatted milk powder) was added and incubated at 37°C for 2 h. Next, 100 μL of serum sample was incubated at 25°C for 2 h, and then the microtiter plate was washed 3 times with PBST for 5 min each. Subsequently, 100 μL of HCP or pHSA or OsrHSA conjugated with biotin (5 μg/mL) was added and incubated at 25°C for 1 h and subsequently washed 6 times with 300 μL of PBST for 5 min each. Next, 100 μL of streptavidin-HRP (1:2,000 dilution) was added and incubated at 25°C for 1 h. Then, 100 μL of chromogenic agents was added after the microtiter plate was washed 6 times for 5 min each. Microtiter plates were incubated at 25°C for 5 min, and then 100 μL of termination solution was added. The antibody titer was measured with a microtiter plate reader.

### Statistical analysis

All data were collected via the Provantis System (SAS 9.2 statistical software) and Microsoft Office EXCEL. One-way ANOVA was used for further analysis if the results did not reach statistical significance (*P*>0.05). If the results of the one-way ANOVA analysis did not reach statistical significance (*P*>0.05), then Dunnett’s test was used for further analysis.

## Results

### The clinical observations

During the experimental period, neither animal death nor abnormal reactions were observed in the HCP and the negative control groups. However, abnormal responses, including transient foaming, shortness of breath sounds, and reduction of activity were occasionally observed in a few female animals in the pHSA and OsrHSA groups but were not found in male animals (S1 Table). This finding could be due to individual differences among the animals. However, no significant difference was obtained between the OsrHSA and pHSA groups.

The body weights of all groups increased during the treatment period except at Day 42. The trend in increasing weights of the male animals was much greater than that of female animals. There was no significant change in the body weight in the HCP group compared with that in the negative control throughout the experimental period. However, a significant difference was observed in female animals in the pHSA group at Day 15 compared with that in the negative control group (*P*<0.05) (S2 Table), and no significant changes were found between the pHSA and OsrHSA groups.

### The HCPs and OsrHSA did not show immunotoxicity

**Fig 1:**
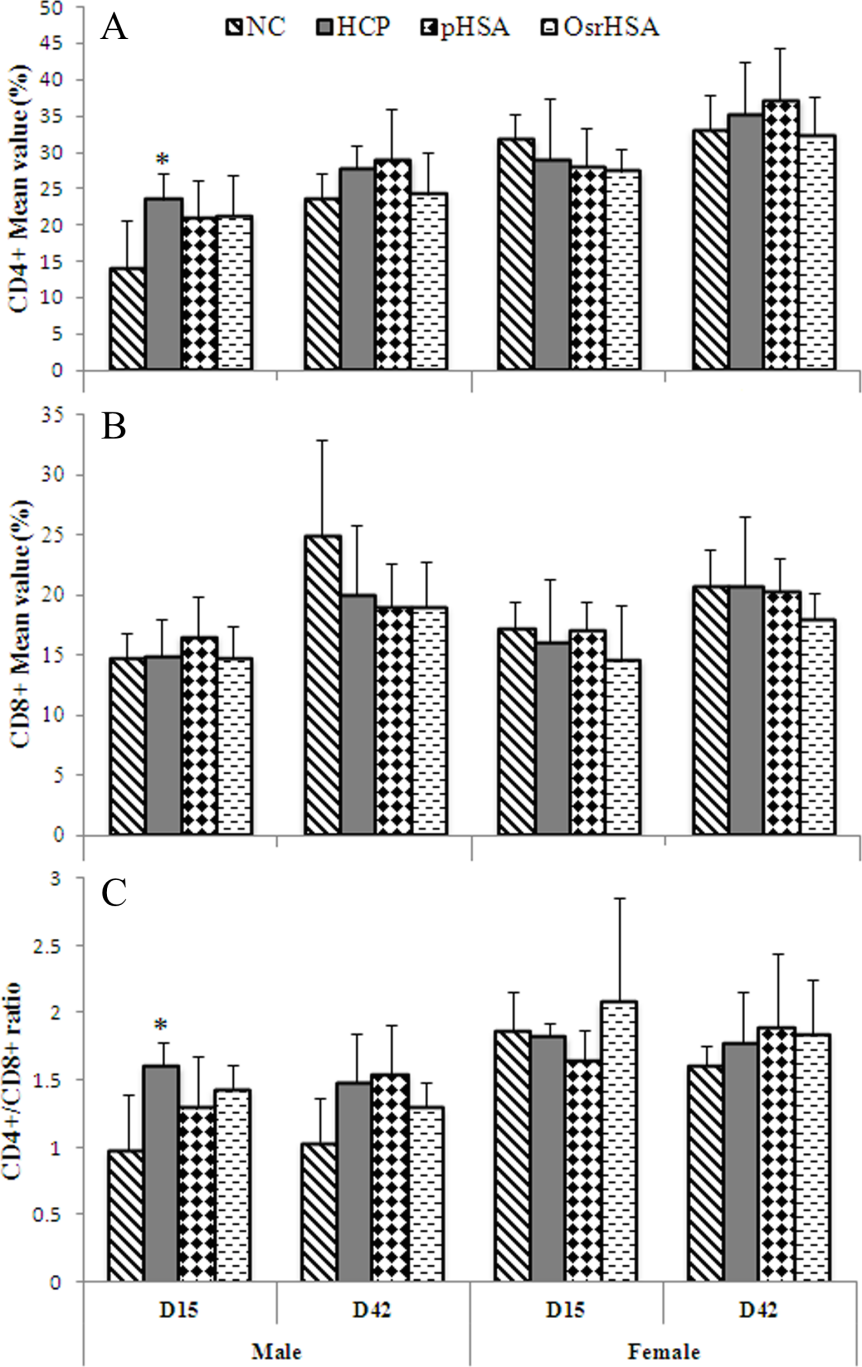
The changes in T-lymphocyte subsets at D15 and D42. Panel A shows CD4+ T cells, panel B shows CD8+ T cells, and panel C shows the ratio of CD4+/CD8+ T cells. The data are presented as the mean ± SD (n = 5). "*" Indicates a statistically significant difference according to ANOVA and Dunnett’s test (*P*≤0.05).

CD4^+^ and CD8^+^ T cells are key subsets regulating diverse immune reactions [31]. Activated T cells differentiate into cytotoxic, helper or regulatory T cells [32]. Therefore, monitoring the proliferation of lymphocytes is one of the important measures for assessing the potential immunotoxicity of biopharmaceuticals. In the present study, CD4^+^ and CD8^+^ T cells were monitored. As shown in Fig 1A and 1B, at Day 15, there were significantly more CD4+ T cells in the HCP, OsrHSA and pHSA groups than those in the negative control, but no significant difference among the HCP, OsrHSA and pHSA groups was observed. CD8^+^ T cells did not exhibit significant differences in the HCP group compared with those in the negative control. However, the ratio of CD4^+^/CD8^+^ T cells at Day 15 showed significant differences in the HCP, OsrHSA, and pHSA groups compared with that in the negative control (Fig 1C), while no significant difference among the HCP, OsrHSA, and pHSA groups was observed. Although the ratio of CD4^+^/CD8^+^ at Day 15 showed a significant difference, the percentage of lymphocyte cells was in the normal clinical range. Furthermore, these changes did not exhibit correlations between drug dose and administration time. Therefore, these changes were not considered immunotoxicity related to this drug.

Cytokines are a group of immunomodulating proteins produced by a wide range of cells. High cytokine levels will over-activate the immune system and cause a life-threatening cytokine storm [33]. Therefore, monitoring cytokine levels can assess or predict the immunotoxicity of biopharmaceuticals [24,34]. A group of cytokines highly related to inflammation, IL-4, IL-5, IL-6, IL-10, IL-13, TNF-α, IFN-γ, and IL-1β, was investigated. The results indicated that none of these eight cytokines were detected in any of the treatment groups (S3 Table). These results suggested that the HCPs from OsrHSA and OsrHSA had no immunotoxicity.

### The HCPs showed low immunogenicity

To determine whether specific antibodies against pHSA, HCPs, and OsrHSA were developed after administration, the titer and incidence of specific antibodies were monitored at Day 14, Day 28 and Day 41. No specific antibodies were detected against OsrHSA, pHSA, and HCPs in the HCP treatment at Day 14 and Day 28. Specific antibodies against the OsrHSA were detected in only 1/10 of the animals at Day 41, but the titer was less than 1.0. Antisera against pHSA were detected in 1/10 of the animals with a titer of 2.0. Antisera against the HCPs were detected in 2/10 of animals, and the titer was less than 1.0. Although specific antisera were detected, the incidence and the titer were low, suggesting that the HCPs have low immunogenicity (Table 1).

**Table 1:** The titers and incidence of antibodies against OsrHSA, pHSA and HCP.

DAT	Group	Anti-OsrHSA-specific antibodies		Anti-pHSA-specific antibodies		Anti-HCP-specific antibodies	
		Incidence	Titer	Incidence	Titer	Incidence	Titer
D14	NC^b^	0/10	ND^a^	0/10	ND^a^	0/10	ND^a^
	HCP	0/10	ND^a^	0/10	ND^a^	0/10	ND^a^
	pHSA	10/10	4~32	10/10	2~64	0/10	ND^a^
	OsrHSA	8/10	<1~512	10/10	<1~256	1/10	<1.0
D28	NC^b^	0/10	ND^a^	0/10	ND^a^	0/10	ND^a^
	HCP	0/10	ND^a^	0/10	ND	2/10	<1.0
	pHSA	10/10	16~256	9/10	16~256	0/10	ND^a^
	OsrHSA	10/10	4~256	10/10	2~256	2/10	<1
D41	NC^b^	0/10	ND^a^	3/10	<1.0	0/10	ND^a^
	HCP	1/10	<1.0	1/10	<2	2/10	<1.0
	pHSA	10/10	<2~1024	10/10	<2~2048	0/10	ND^a^
	OsrHSA	10/10	16~1024	10/10	64~2048	3/10	<1.0

Note

^a^ “ND”: Not detected. Data are expressed as the number of incidences/total animals.

^b^ NC = Negative control.

The antibodies against OsrHSA, pHSA, and HCPs in pHSA and OsrHSA treatments were examined. As shown in Table 1, the incidences of antisera against OsrHSA were 10/10 (pHSA) and 8/10 (OsrHSA), and the titers of antisera ranged from 4–32 (pHSA) and <1–512 (OsrHSA) at Day 14. The incidence of antisera against pHSA was 10/10 (pHSA) and 8/10 (OsrHSA), and the antisera titer ranged from 2–64 (pHSA) and <1–256 (OsrHSA). The incidence of antisera against HCPs was 0/10 (pHSA) and 1/10 (OsrHSA), and the antisera titer was less than 1.0. The incidence and the titers of antisera against OsrHSA and pHSA notably differed at Day 14. The highest titers of 256 were developed at Day 28, while the highest titers of 1,024 (anti-OsrHSA) and 2,048 (anti-pHSA) were developed at Day 41 (Table 1). In particular, the titer of anti-HCP antibodies was less than 1.0, consisting of the results of the HCPs alone, again demonstrating that the HCPs showed low immunogenicity.

### The HCPs and OsrHSA did not promote immune responses

**Fig 2:**
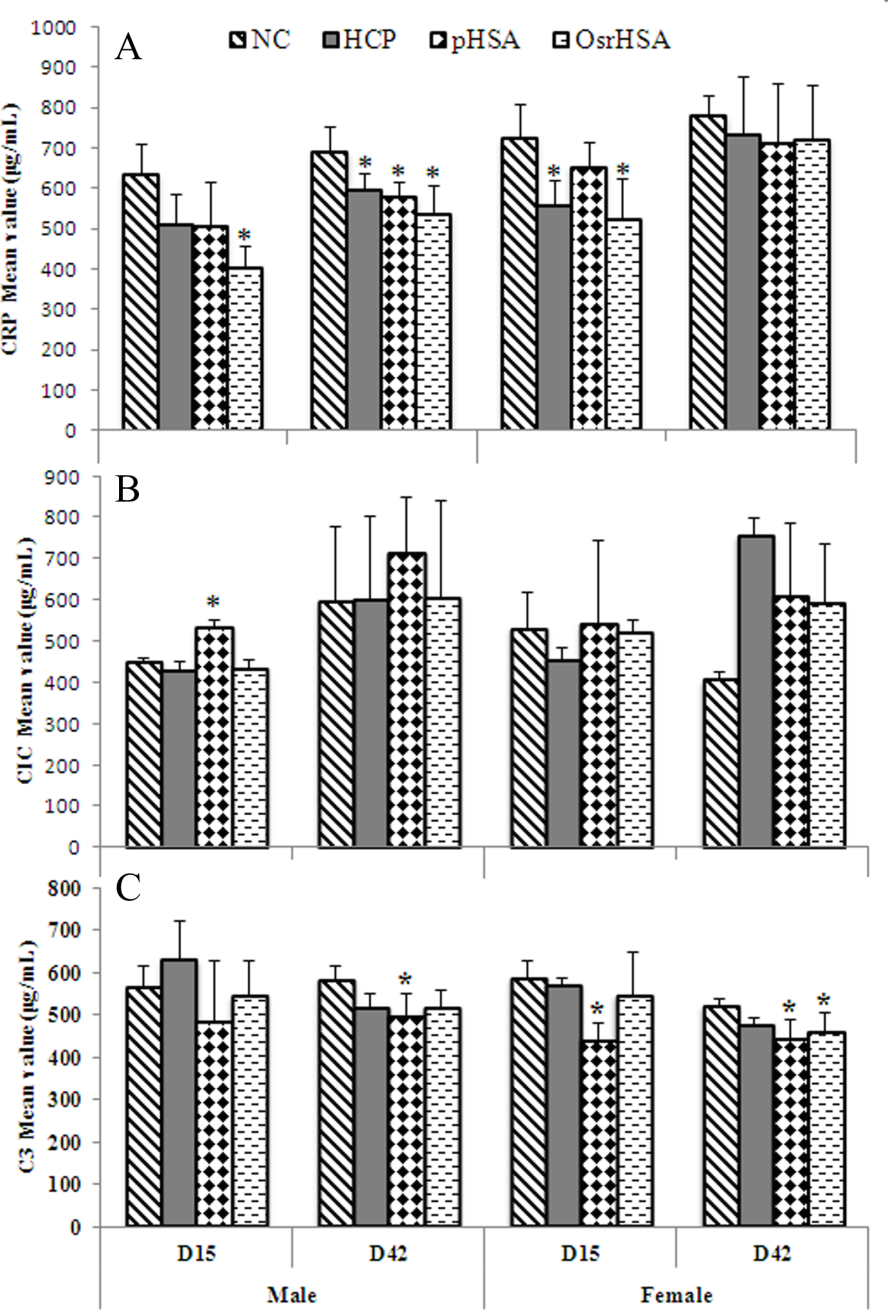
The changes in CRP, CIC and C3 levels at D15 and D42. Panel A is CRP, panel B is CIC, and panel C is C3. Data are presented as the mean ± SD (n = 5); "*" Indicates a statistically significant difference according to ANOVA and Dunnett’s test (*P*≤0.05).

The expression of CRP, an acute-phase protein of hepatic origin, increases following interleukin-6 secretion from macrophages and T-lymphocytes and subsequently CRP activates the complement system via the C1Q complex [35]. The increase in the CRP level in plasma indicates the start of an inflammatory process. As shown in Fig 2A, CRP did not increase but, rather, significantly decreased in male animals in the OsrHSA group at Day 15 and in the HCP, pHSA and OsrHSA groups at Day 42 (*P*<0.05). Although CRP significantly decreased in the HCP and pHSA groups of female animals at D15 (Fig 2A), no correlation with drug and administration time course was observed.

The CIC is a normally protective immune system. CIC formation is a natural response to eliminate antigens in serum circulation. ADAs are the result of unwanted immunogenicity reactions, serving as the immune responses of an organism against a therapeutic drug. Regardless of the cause of immunogenicity, an immunogenic response in the form of ADAs induces a humoral immune response. ADAs inactivate the effects of therapeutics, binding to biopharmaceuticals in the circulation to form immune complexes [36,37]. Increases in CIC levels are important criteria for developing ADAs. To assess the ADAs of the HCPs and OsrHSA, we monitored the level of CIC at Day 15 and Day 42. The results showed that no significant drug-related CIC change was observed in the HCP and OsrHSA groups compared with that in the negative control (*P*>0.05), although the CIC in male animals was significantly increased in the pHSA group at Day 15 (*P*≤0.05) (Fig 2B). However, this increase did not exhibit a correlation with the administration time.

C3 and C4 are immune proteins that play a central role in the complement system and contribute to innate immunity [38]. The activation of immune reactions is critical when the immune system recognizes antigens, such as the HCPs in this case. No significant changes in C3 levels in the HCP group at Day 15 and Day 42 were observed compared with those in the negative control (Fig 2C). The C3 level in the pHSA group was significantly decreased at Day 15 and Day 42 but decreased in female OsrHSA animals at Day 42 (*P*>0.05) (Fig 2C). In addition, C4 levels were not detected in any treatment groups.

### The pathological changes of OsrHSA could be recovered

Due to the high dose of OsrHSA infused, pathological changes occurred as expected. We observed several main vital organs. We did not find significant changes in organ weights and coefficient ratios in the HCP group. Furthermore, no significant drug-related pathological changes were observed between the HCP group and the negative control, including liver cell hypertrophy, liver/splenic hematopoiesis, glomerular mesangial cell proliferation and matrix enlargement, tubular tubule and renal tubular degeneration/regeneration (Table 2).

**Table 2:** The incidence of pathological changes in target organs in different treatments at D15 and D42.

Time	Pathological changes	NC^b^		HCP		pHSA		OsrHSA	
		Male	Female	Male	Female	Male	Female	Male	Female
D15	Hepatocyte hypertrophy	0/10	0/10	0/10	0/10	4/10	3/10	6/10	3/10
	Liver extra medullary hematopoiesis	0/10	0/10	0/10	0/10	3/10	2/10	5/10	0/10
	Splenic hematopoiesis	1/10	0/10	1/10	0/10	9/10	4/10	7/10	5/10
	Tubular tubule type	2/10	0/10	0/10	0/10	6/10	9/10	7/10	9/10
	Renal tubular degeneration/ regeneration	2/10	0/10	0/10	0/10	7/10	8/10	10/10	9/10
	Glomerular mesangial/hyperplasia and increased matrix	0/10	0/10	0/10	0/10	10/10	10/10	10/10	10/10
D42	Hepatocyte hypertrophy	ND^a^	ND^a^	ND^a^	ND^a^	ND^a^	ND^a^	ND^a^	ND^a^
	Liver extra medullary hematopoiesis	ND^a^	ND^a^	ND^a^	ND^a^	ND^a^	ND^a^	ND^a^	ND^a^
	Splenic hematopoiesis	ND^a^	ND^a^	ND^a^	ND^a^	ND^a^	ND^a^	ND^a^	ND^a^
	Tubular tubule type	1/5	0/5	0/5	0/5	1/5	4/5	1/5	5/5
	Renal tubular degeneration/ regeneration	0/5	0/5	0/5	0/5	3/5	4/5	4/5	4/5
	Glomerular mesangial/hyperplasia and increased matrix	0/5	0/5	0/5	0/5	5/5	5/5	5/5	5/5

Note

^a^ “ND”: Not detected.

^b^ NC: Negative control.

Drug-related pathological changes in the pHSA and the OsrHSA groups were observed, including the increase of the organ-to-body weight ratios of the liver, spleen, and kidneys at Day 15, which could be associated with overloading from the high-dosage infusion of the proteins. However, no difference in the organ weights and coefficient ratios was observed between the HCP group and the negative control and pHSA and OsrHSA groups (S4 Table). To further analyze whether precancerous lesions formed in pHSA and OsrHSA treatments, histochemical observation of the pathological changes in organs was performed. Compared with the negative control, the precancerous lesions from hepatocyte hypertrophy, liver extramedullary hematopoiesis, splenic hematopoiesis, glomerular mesangial cell incrassation, hyperplasia/increased matrix, and tubular tubule type and renal tubular degeneration/regeneration were observed at Day 15. The precancerous lesions were highly drug related because of the high dose of pHSA and the OsrHSA. However, no drug-related precancerous lesions were observed for the liver and spleen at Day 42, indicating that the precancerous lesions were completely recovered. No obvious differences in the incidence and degree of precancerous lesions were observed between the pHSA and OsrHSA groups (Table 2).

**Fig 3:**
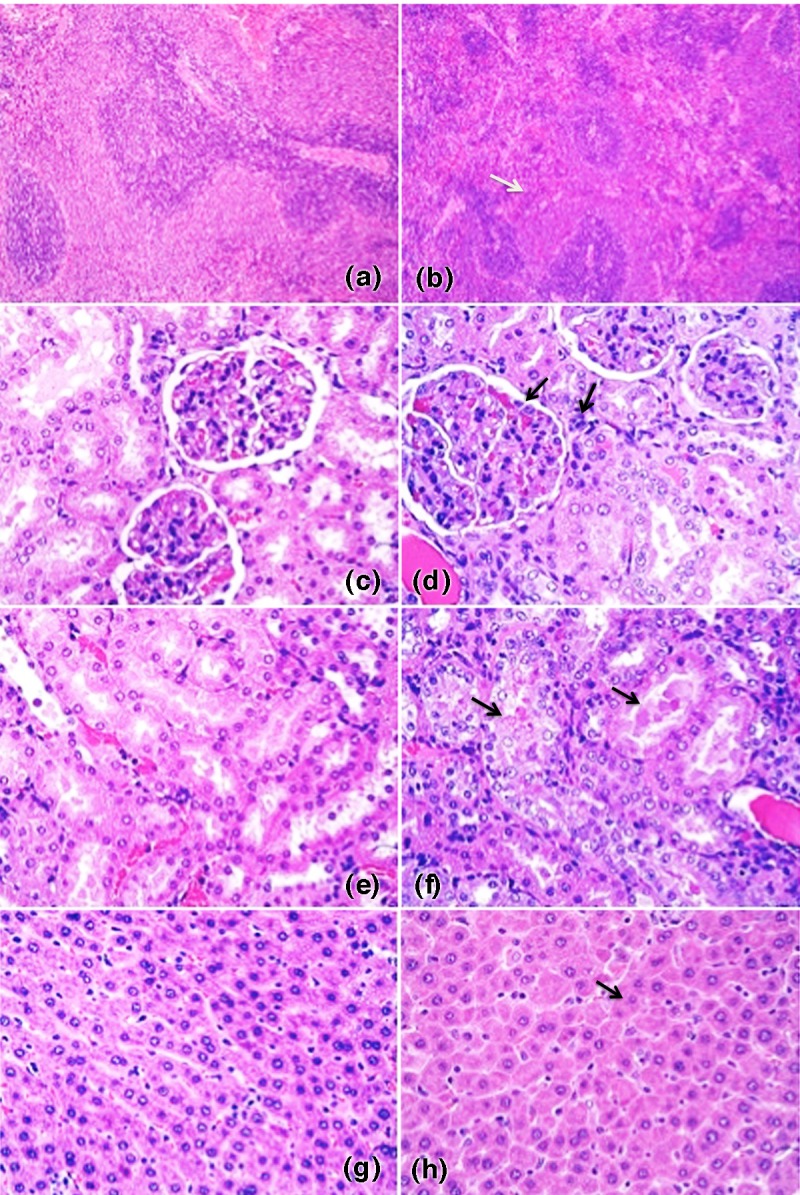
The histogram of the pathological observations of spleen, kidney, and liver tissues in OsrHSA. Panels (A) and (B) show diagrams of the spleen. The extramedullary hematopoiesis in the OsrHSA treatment (B) was visible compared with the negative control (A); (C) to (F) show diagrams of the kidney tissue. Obvious glomerular mesangial and renal matrix hyperplasia in OsrHSA (D) was observed compared with the negative control (C). Renal tubular and renal tubular degeneration/regeneration in OsrHSA (F) was visible compared with the negative control (E). No changes in hepatitis cell infiltration of the liver were observed compared to the negative control. The extramedullary hematopoiesis of liver was observed in the OsrHSA group (H) compared with the negative control (G). The magnification is 10x in (A) and (B) and 40x in (C) to (H).

Obvious visible precancerous lesions were observed in the liver, kidney, and spleen at Day 15, showing the drug-related precancerous lesions of extramedullary hematopoiesis in the spleen (Fig 3A vs 3B), glomerular mesangial and renal hyperplasia in the kidneys (Fig 3C vs 3D), renal tubular and degeneration or regeneration of renal tubules in the kidneys (Fig 3E vs 3F) and hepatitis cell infiltration in the liver (Fig 3G vs 3H). However, these precancerous lesions were obviously alleviated or recovered at Day 42, presenting the recovery trends of the observed precancerous lesions. No difference between the OsrHSA and the pHSA groups was found, indicating that the precancerous lesions could be attributed to the high dose of the pHSA or OsrHSA via the increased osmotic pressure and aggravated in the kidney.

## Discussion

OsrHSA, administered at a high dosage in the clinic, was the first PMP approved for clinical trials in China. Because OsrHSA is biochemically and physically identical to pHSA [5], the safety concern of OsrHSA does not come from HSA itself but from the residual HCPs and other impurities produced during OsrHSA processing. Although the HCP impurities in OsrHSA are controlled to concentrations as low as 1.5 μg/g, the total HCP content is still high because HSA is occasionally administered at more than 50 g/dose in the clinic. Thus, it is essential to obtain direct evidence of the immunotoxicity of residual HCPs in OsrHSA through preclinical trial/animal systems. In the present study, we used an animal system to assess the immunogenicity of the HCPs and OsrHSA in parallel. The results indicated that OsrHSA and HCP treatments exhibited low immunotoxicity and that the 25 μg/kg dosage of HCPs equivalent to a 25-fold dose of OsrHSA or 150 g/dose is safe in humans. Furthermore, these data indicated that the residual HCPs of OsrHSA do not produce immunotoxicity. Although general immunotoxicity-related responses were observed in both the pHSA and OsrHSA groups, no significant differences were found between these groups, suggesting that OsrHSA is as safe as pHSA.

We further did not observe any differences in the immunogenicity between pHSA and OsrHSA. In particular, no significant differences in T-lymphocyte cell subsets, CD4^+^, CD8^+^ T cells and the CD4^+^/CD8^+^ ratio were observed between the pHSA and OsrHSA groups. Moreover, no drug-related immunological changes in CRP, CIC, C3, or C4 between the pHSA and OsrHSA groups were detected. Although OsrHSA-, pHSA- and HCPs-specific antibodies were developed in the pHSA and OsrHSA treatments, the titer was low, close to the background levels. In addition, we did not detect IgE or IgM titers during the entire experimental period. Furthermore, our results excluded the potential risk of a “cytokine storm” [33]. These results are consistent with those of a recent study using human PBMCs to predict cytokine storms and the immunotoxicity of OsrHSA [24].

In general, the severity of the immunogenicity for HCPs is far greater for microbial organisms than for mammals, rodents, and plants. Rice has been a staple crop for thousands of years. We hypothesize that human beings have developed high tolerance to rice proteins during evolution. In the present study, we confirmed the hypothesis that the residual HCPs from rice are safe in humans. Therefore, the seeds of cereal crops have great potential as hosts or target organs for molecular pharming [33]. Although the seeds of other cereal crops, such as wheat, barley, and maize, could be used as hosts for molecular pharming, the storage protein glutelin from wheat and barley seeds is a potential allergen that causes an autoimmune disease called celiac disease. Therefore, these seeds are not suitable hosts for plant molecular pharming. Maize is also recognized as an excellent host, but this plant is a cross-pollinating crop with considerable challenges with respect to biosafety. Thus, rice seed may be the best choice in terms of self-pollinating crops, and its storage proteins produce no known allergic reactions.

## Supporting information

S1 Table Clinical observations of the HCP, OsrHSA and pHSA groups. (DOCX)

S2 Table Body weight in the OsrHSA, HCP and pHSA groups at different time points. (DOCX)

S3 Table The level of cytokines on D15 and D42. (DOCX)

S4 Table Organ-to-body weight ratio in the OsrHSA, HCP and pHSA groups. (DOCX)
