# Supplementary material for: Assessment of the immunogenicity of residual host cell protein impurities of OsrHSA
Source: PLoS One. 2018 Mar 7;13(3):e0193339. doi: 10.1371/journal.pone.0193339 (PMC5841786; doi:10.1371/journal.pone.0193339)
Supplement: S1 Table — (DOCX) [file pone.0193339.s001.docx]

# Supporting information

**S1 Table. Clinical observations among the HCP, OsrHSA and pHSA groups**

| Phenotype | |  |  |  |  |  |  |  |  |
| --- | --- | --- | --- | --- | --- | --- | --- | --- | --- |
|  |  | NC | | HCP | pHSA | | OsrHSA | |  |
| Lassitude | Male | | 0/15 | 0/15 | | 0/15 | | 0/15 | |
|  | Female | | 0/15 | 0/15 | | 1/15 | | 0/15 | |
| Spit foaming | Male | | 0/15 | 0/15 | | 0/15 | | 0/15 | |
|  | Female | | 0/15 | 0/15 | | 2/15 | | 1/15 | |
| Shortness breath sounds | Male | | 0/15 | 0/15 | | 0/15 | | 0/15 | |
|  | Female | | 0/15 | 0/15 | | 1/15 | | 1/15 | |
| Activity reduction | Male | | 0/15 | 0/15 | | 0/15 | | 0/15 | |
|  | Female | | 0/15 | 0/15 | | 1/15 | | 0/15 | |

Note: NC: Negative Control; Data are expressed as the number of incidences per total animals.
