# Supplementary material for: Assessment of the immunogenicity of residual host cell protein impurities of OsrHSA
Source: PLoS One. 2018 Mar 7;13(3):e0193339. doi: 10.1371/journal.pone.0193339 (PMC5841786; doi:10.1371/journal.pone.0193339)
Supplement: S2 Table — (DOCX) [file pone.0193339.s002.docx]

# Supporting information

**S2 Table. Body weights in the OsrHSA, HCP and pHSA groups at different time points**

| Treatments Sex | | D7 | D15 | D28 | D35 | D42 |
| --- | --- | --- | --- | --- | --- | --- |
| NC | Male | 265.4±12.2 | 294.2±18.0 | 379.0±46.9 | 408.4±54.7 | 392.6±56.4 |
|  | Female | 205.9±10.7 | 205.6±8.8 | 270.8±25.3 | 271.8±14.6 | 257.4±15.9 |
| HCP | Male | 263.8±10.0 | 287.7±13.1 | 388.2±25.3 | 414.2±26.2 | 398.6±27.9 |
|  | Female | 203.7±8.4 | 202.7±6.8 | 240.8±13.7 | 248.2±12.7 | 237.0±6.9 |
| pHSA | Male | 256.9±9.1 | 292.2±14.5 | 357.6±23.6 | 381.6±28.8 | 364.8±28.6 |
|  | Female | 208.5±6.2 | 215.9*±8.3 | 254.6±17.6 | 257.2±23.6 | 245.6±21.0 |
| OsrHSA | Male | 260.5±10.7 | 293.3±11.5 | 277.0±41.6 | 404.2±44.4 | 387.6±39.9 |
|  | Female | 203.5±8.8 | 213.9± 8.8 | 244.8±32.1 | 250.2±26.8 | 235.6±19.2 |

Note: NC: Negative Control . Data are expressed as the mean ± SD (n=10). "*" indicates a statistically significant difference (*P*≤0.05).
