# Supplementary material for: Assessment of the immunogenicity of residual host cell protein impurities of OsrHSA
Source: PLoS One. 2018 Mar 7;13(3):e0193339. doi: 10.1371/journal.pone.0193339 (PMC5841786; doi:10.1371/journal.pone.0193339)
Supplement: S3 Table — (DOCX) [file pone.0193339.s003.docx]

# Supporting information

**S3 Table. The level of cytokines on D15 and D42**

| Group | Sex | IL-4 | IL-5 | IL-6 | IL-10 | IL-13 | TNF-α | IFN-γ | IL-1β |
| --- | --- | --- | --- | --- | --- | --- | --- | --- | --- |
| NC^a^ | Male | ND^b^ | ND^b^ | ND^b^ | ND^b^ | ND^b^ | ND^b^ | ND^b^ | ND^b^ |
|  | Female | ND^b^ | ND^b^ | ND^b^ | ND^b^ | ND^b^ | ND^b^ | ND^b^ | ND^b^ |
|  | Male | ND^b^ | ND^b^ | ND^b^ | ND^b^ | ND^b^ | ND^b^ | ND^b^ | ND^b^ |
| HCP | Female | ND^b^ | ND^b^ | ND^b^ | ND^b^ | ND^b^ | ND^b^ | ND^b^ | ND^b^ |
|  | Male | ND^b^ | ND^b^ | ND^b^ | ND^b^ | ND^b^ | ND^b^ | ND^b^ | ND^b^ |
| pHSA | Female | ND^b^ | ND^b^ | ND^b^ | ND^b^ | ND^b^ | ND^b^ | ND^b^ | ND^b^ |
|  | Male | ND^b^ | ND^b^ | ND^b^ | ND^b^ | ND^b^ | ND^b^ | ND^b^ | ND^b^ |
| OsrHSA | Female | ND^b^ | ND^b^ | ND^b^ | ND^b^ | ND^b^ | ND^b^ | ND^b^ | ND^b^ |

Note: NC^a^: Negative Control; ND^b^: Not detected (n=6).
