# Supplementary material for: Assessment of the immunogenicity of residual host cell protein impurities of OsrHSA
Source: PLoS One. 2018 Mar 7;13(3):e0193339. doi: 10.1371/journal.pone.0193339 (PMC5841786; doi:10.1371/journal.pone.0193339)
Supplement: S4 Table — (DOCX) [file pone.0193339.s004.docx]

# Supporting information

**S4 Table. Organ-to-body weight ratios in the OsrHSA, HCP and pHSA groups**

| Treatments Sex | | Organ-to-body weight ratios (%) | | | | | | | |
| --- | --- | --- | --- | --- | --- | --- | --- | --- | --- |
|  |  | Heart | | Liver | | Spleen | | Kidney | |
|  | | D15 | D42 | D15 | D42 | D15 | D42 | D15 | D42 |
| NC | Male | 0.43±0.03 | 0.41±0.05 | 3.04±0.22 | 2.59±0.17 | 0.23±0.03 | 0.17±0.02 | 0.82±0.06 | 0.67±0.03 |
|  | Female | 0.46±0.06 | 0.41±0.03 | 2.96±0.16 | 2.61±0.14 | 0.22±0.04 | 0.22±0.02 | 0.74±0.06 | 0.65±0.04 |
| HCP | Male | 0.46±0.03 | 0.41±0.05 | 3.05±0.24 | 2.65±0.17 | 0.27±0.04 | 0.18±0.02 | 0.81±0.04 | 0.73±0.05 |
|  | Female | 0.46±0.06 | 0.43±0.04 | 2.87±0.14 | 2.62±0.31 | 0.24±0.03 | 0.22±0.03 | 0.74±0.07 | 0.71±0.06 |
| pHSA | Male | 0.47±0.04 | 0.40±0.02 | 3.42*±0.12 | 2.66±0.27 | 0.27±0.06 | 0.19±0.02 | 0.87±0.04 | 0.74±0.05 |
|  | Female | 0.44±0.03 | 0.43±0.04 | 3.48*±0.24 | 2.74±0.21 | 0.29*±0.05 | 0.24±0.03 | 0.96*±0.12 | 0.83*±0.10 |
| OsrHSA | Male | 0.46±0.06 | 0.40±0.03 | 3.61*±0.19 | 2.82±0.24 | 0.30*±0.05 | 0.20±0.01 | 0.88*±0.06 | 0.77*±0.05 |
|  | Female | 0.46±0.04 | 0.41±0.02 | 3.52*±0.28 | 2.83±0.15 | 0.29*±0.05 | 0.26±0.03 | 0.96*±0.24 | 0.87*±0.15 |

Note: NC: Negative Control
